# Floristic Composition, Structure, and Regeneration Status of Woody Plants in Wonjeta St Micheal Church Forest, Northwestern Ethiopia

**DOI:** 10.1155/2023/4061029

**Published:** 2023-04-20

**Authors:** Amare Bitew Mekonnen, Wubetie Adnew Wassie

**Affiliations:** Bahir Dar University, Department of Biology, Bahir Dar, Ethiopia

## Abstract

The study was conducted in a historian Wonjeta St Michael Church Forest, believed to be constructed in 11^th^ century in Northwestern Ethiopia. The composition, structure, and management of this forest is not documented. Fifty plots of 20 m × 20 m (400 m^2^) were laid along five-line transect for vegetation data collection. In addition, 5 m × 5 m subplots were laid within the main plot to sample seedlings and saplings. All plots were laid at a distance of 50 m along the transect lines. The diversity and population structure of woody individuals of trees and shrubs with a diameter at breast height (DBH) ≥ 2.5 cm and height ≥2 m were measured, and DBH <2.5 cm and height <2 m were counted as seedlings and saplings. All trees and shrubs recorded in the 50 plots were used for vegetation structure analysis. A total of 65 woody plant species in 53 genera and 33 families were recorded. Out of the total number of species, three were found to be endemic to Ethiopia. The family Fabaceae had the highest number of species, followed by Moraceae, and then Euphorbiaceae with 14, 6, and 4 species, respectively. The results of Shannon Wiener diversity and evenness indices of woody species were 2.8 and 0.68, respectively. Woody species densities for mature individuals were 2,202.5 stems ha^−1^, seedling 2419.2 stems ha^−1^, and sapling 1737.6 stems ha^−1^. Priority for conservation should be given using population structure, important value index, and regeneration status as criteria. Results of the structural analysis revealed that the Forest is highly dominated by small-sized trees and shrubs indicating that it is in the stage of secondary development and there are species that require urgent conservation measures.

## 1. Introduction

Tropical forests are biologically diversified ecosystems with high species richness, evenness, net primary production (NPP), and biomass accumulation due to prevailing favorable environmental conditions[1]. They are, however, highly threatened by anthropogenic activities [2]. Deforestation is still alarmingly high in the tropical region resulting formation of small patches of fragmented forests [3]. Loss of forest cover and biodiversity due to anthropogenic activities is a growing concern in many parts of the world. Africa's forest cover is estimated to be 650 million hectares constituting 17% of the world's forests including several global biodiversity hotspots [4].

Ethiopia is one of the top 25 biodiversity-rich countries in the world and hosts two of the world's biodiversity hotspots, namely, the Eastern Afromontane and the Horn of Africa hotspots. It is also among the countries in the Horn of Africa regarded as the chief Centre of diversity and endemism for several plant species. The Ethiopian flora is estimated to have about 6000 species of higher plants, of which 10% have considered to be endemic [5]. Woody plants constitute about 1000 species [6]. This richness in biodiversity is due to topographical diversity with flat-topped plateaus, high mountains, river valleys, deep gorges, rolling plains, and variation of altitude ranging from 116 to 4620 meter above sea level (m.a.s.l.) [7]; (IBC, 2014).

Many scholars also agree that the forest of Ethiopia is decreasing from time to time due to anthropogenic activities [8, 9]; lack of integration of the local people living around the conservation areas is the major constraint to the overall conservation effort in Ethiopia [10]. Due to this, it has now realized that unless the local community is involved in the conservation effort, the sustainability of forest resources will be under question. Rapid human population growth, poverty, forest clearing for cultivation, overgrazing, exploitation of forests for fuel wood, and construction materials without replantation and lack of proper policy framework are some of the factors that contribute to the loss of forest resources in Ethiopia [11, 12]. Reduction in forest cover has several consequences, such as soil erosion and reduced capacity for watershed protection with possible flooding, reduced capacity, and loss of biodiversity. This leads to the instability of the ecosystem and reduced availability of various forest products and services [13].

Ethiopia's northern and central highlands are nearly devoid of forests due to deforestation and environmental degradation, where forests remain as small, isolated patches always on the tops of mountains and heads of streams surrounding churches [14]. These remnant natural forest fragments persist because of their sacred status under religious knowledge of forest conservation of the Ethiopian Orthodox Tewahido Churches (EOTC) [14, 15].

Wonjeta St Michael Church Forest is one of the few reminant natural forests in the Ethiopian upper Blue Nile basin, where most of the forest area has degraded and converted to agricultural land due to demographic pressure, sedentary farming, and exotic tree plantation. Despite of all these, Wonjeta St Michael Church Forest is not yet studied. These inspired researchers to study the vegetation status, woody species composition, density, diversity, vegetation structures, and regeneration status of the forest. Therefore, this study aimed to describe the opportunities and challenges of such Church forest for the upper Blue Nile basin and ecosystem wellbeing.

## 2. Materials and Methods

### 2.1. Description of the Study Area

**Figure 1:**
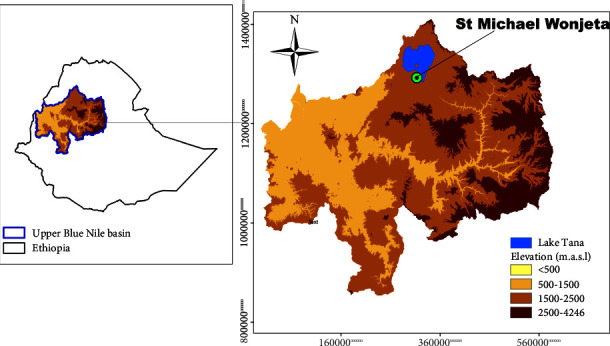
The study area map.

The study was conducted in Wonjeta St Michael Church Forest, Bahir Dar zuria District in Ethiopian upper Blue Nile basin, Northwestern Ethiopia (Figure 1). Bahir Dar zuria is one of the districts in West Gojjam administrative Zone. The district is bounded in the south by Yelimanadensa District, on the southwest by Mecha, on the northwest by GilgelAbay River (tributary) separates it from Semien Achefer, on the north by Lake Tana, on the east by the Abay River that separates it from South Gonder Zone, and in the west by Mecha District and Achefer District. The study site is Wonjeta St Michael Church Forest which is found in the reference of Longitude 11.686410 and Latitude 37.283097 with the elevation of 1820 m.a.s.l.

### 2.2. Methods of Sampling and Data Collection

#### 2.2.1. Sampling Design

Systematic sampling design was used to collect vegetation data following Muller and Ellenberg [16].

Five-line transects were laid parallel to each other and 50 m apart.

The first line transect was started 50 meters inward from the edge of the forest to avoid “edge effect” [17, 18]. Along each transect, sample plots having an equal size of 20 m × 20 m (400 m^2^) were laid at distance of 50 m from each other. A total of 50 plots were laid for woody species data collection. Within the main plot, 5 m × 5 m subplots were laid at four corners and one in the middle for seedling and sapling data collection.

#### 2.2.2. Vegetation Identification and Data Collection

All woody plant species encountered in each plots were identified and recorded with structural and abundance data. Identification of those plant species were carried out by their local (vernacular) name using experienced key informants and taxonomist. For those species difficult to identify in the field, their voucher specimens were collected, and the specimens were brought to Bahir Dar University herbarium for taxonomic identification and by referring published volume of the flora of Ethiopian and Eritrea [19–21].

#### 2.2.3. Structural Data Collection

The diameter at breast height (DBH) and height of woody plants was recorded with measuring tape and calibrated stick, respectively, which were used for description of vegetation structure (frequency, dominancy, basal area, and important value index).

In each plot, trees and shrubs with height ≥2 m and DBH ≥ 2.5 cm were measured and recorded at about 1.35 m from the ground. For trees and shrubs that were branched around the breast height, the circumference was measured separately and averaged. For the purpose of regeneration assessment, from the five subquadrats, the seedlings and saplings were counted for each species. In this study, a seedling was considered as woody individual with a DBH < 2.5 cm and less than 1 m height; a sapling was considered as those woody individual with a DBH ≥ 2.5 cm and a height of less than 2 m.

### 2.3. Data Analysis

#### 2.3.1. Vegetation Composition and Structure Data Analysis

The structural parameters were analyzed using the following formula:

Basal area of a tree = *π*(DBH/2)^2^ Where *π* = 3.142, DBH (diameter at breast height) = (C/*π*).

(1) $$\begin{array}{c} Dominance=\frac{Basal\;area\;of\;a\;species}{Area\;sampled}\text{,}\\ Relative\;dominance=\frac{Dominance\;for\;species\;A}{Total\;dominance\;of\;all\;species}\text{,}\\ Density\;\left(D\right)=\frac{Numberofindividuals\;ofa\;species}{Sampled\;area\;in\;ha}\text{,}\\ Relative\;density\;\left(RD\right)=\frac{Number\;of\;Individuals\;of\;a\;species}{Total\;number\;of\;individuals\;in\;ha}x\;100\text{,}\\ Frequency\left(F\right)=\frac{Number\;of\;plots\;in\;which\;a\;species\;occurs}{Total\;number\;of\;plots\;sampled}\text{,}\\ Relativefrequency\left(RF\right)=\frac{frequency\;value\;for\;a\;species}{Total\;frequency\;value\;for\;all\;species}x\;100\text{,}\\ Important\;value=Relative\;density+Relative\;frequency+Relative\;dominance\text{.} \end{array}$$

Importance value index (IVI) analysis is used for setting conservation priority. It combines data for three parameters (relative frequency, relative density, and relative abundance) or it often reflects the extent of the dominance, occurrence, and abundance of a given species in relation to other associated species in an area [22].

The vertical stratification of species in the study area was examined using the IUFRO classification scheme [23]. According to this scheme, tree with >2/3 height of the top represents upper story, trees with height between 1/3 and 2/3 of the top height represent the middle story, and the lower story is represented by trees with height <1/3 of the top height.

#### 2.3.2. Regeneration Data Analysis

Regeneration status of the forest was analyzed by comparing saplings and seedlings with the matured trees according to Dhaulkhandi et al. [24] and Tiwari et al. [25]; i.e., good regeneration, if seedlings > saplings > adults; fair regeneration, if seedlings > or ≤ saplings ≤ adults; poor regeneration, if the species survives only in sapling stage, but no seedlings (saplings may be <, > or = adults); and if a species is present only in an adult form it is considered as not regenerating.

#### 2.3.3. Measurement of Species Diversity Indices

Shannon–Wiener diversity index is used to evaluate the species diversity of the study area. It is popular measure of species diversity and evenness that is not affected by sample size as follows:

(2) $$\begin{array}{c} H^{\prime }=-\overset{S}{\underset{i=1}{\sum }}Pi\left(\ln\left(Pi\right)\right)\text{.} \end{array}$$

H′: Shannon diversity index, *S*:  the number of species, Pi:  the proportion of individual woody plant species, and ln:  is the natural logarism.

The equitability or evenness of the species in each plot was computed using the following formula:

(3) $$\begin{array}{c} Evenness\;index\;\left(Equitability\right)\;J=\frac{H^{\prime }}{H\;\max}\text{,} \end{array}$$

where *J*:  Evenness, H′:  Shannon diversity index, and H′max:  lnS; *S*: the number of species.

Equitability assumes a value between 0 and 1, where 0 indicates the abundance of few species and 1 indicates the condition where all species are equally abundant.

## 3. Result and Discussion

### 3.1. Woody Plant Species Composition

**Figure 2:**
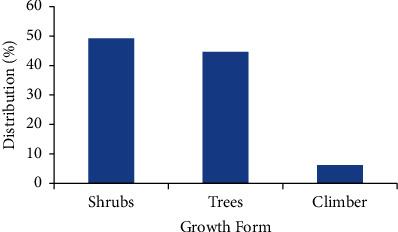
Distribution of woody plant species by their growth form in Wonjeta St Michael Church Forest.

A total of 65 woody plant species in 53 genera and 33 families were identified from Wonjeta St Michael Church Forest. Among the 65 recorded woody species 32 (49.23%) were shrubs, 29 (44.62%) were trees, and 4 (6.15%) lianas (Figure 2). The results of the study revealed that the species richness of the forest was higher than some dry afromontane forests of Ethiopia such as Ambo State Forest with 58 species located in South Gondar Zone [26], Wanzaye natural forest with 49 species in South Gondar [27], and Wogello natural forest 20 species in North Gonder [28] and less than many dry afromontane Forests such as Woynwuha Natural Forest with 69 species in East Gojjam [29] and Chebera Churcura National Park with 106 species in Southern Ethiopia [30].

The major families were Fabaceae represented by 14 species (21.53%) followed by the family Moraceae consisting 6 species (9.23%), and Euphorbiaceae including 4 species (6.15%), Oleaceae with 3 species (4.61%), Asteraceae, Acanthaceae, Celastraceae, Lamiaceae, Apocynaceae, Solanaceae, Malvaceae, Combretacae, and Myrtaceae each with 2 species (3.08% each). Each of the remaining 20 families was represented by one species (30.76%) (Table 1). Different studies [13, 31, 32] reported the dominance of Fabaceae, Poaceae, and Asteraceae in Afromontane vegetation type but it is not true in this study area.

### 3.2. Species Diversity and Evenness

The results of Shannon–Wiener diversity and evenness indices of woody species were 2.8 and 0.68, respectively, which means woody diversity and evenness was high when compare to Yegof forest 2.26 and 0.57, respectively [33]. However, the forest has lower value of diversity and evenness indices than Zegie forest (H′ = 3.72 and *J* = 0.84), respectively [34], and Keja Araba and Tula forest (H′ = 2.81 and 3.14 and *J* = 0.79 and 0.86), respectively [35]. According to Kent and Coker [22], the Shannon-Wiener diversity index normally varies between 1.5 and 3.5 and rarely exceeds 4.5. High diversity when it is above 3.0, medium when it is between 2.0 and 3.0, low between 1.0 and 2.0, and very low when it is smaller than 1. The equitability (evenness) index has values between 0 (a situation in which the abundance of all species are completely disproportional) and 1 (all species are equally abundant) [22]. The study forest has medium diversity may be due to harvesting of fuel wood such as selective cutting, Eucalyptus plantations for timber, or firewood make the forest monocultures leading to homogeneous forests. The clearing of shrubs/trees for agriculture in the study area could also make the forest into isolated small fragments. The main reason for this forest to have medium or low evenness (with a few species dominate) could be because of variable environmental conditions, competition between species, overexploitation of some species, rate of dispersal, growth, and recruitment performances [36]. The diversity and evenness indices imply the need to conserve the forests from human disturbance.

### 3.3. Vegetation Structure

#### 3.3.1. Vegetation Density

The overall density of mature woody species with DBH ≥ 2.5 cm in Wonjeta St Michael Church Forest were 2202.5 stems ha^−1^. This was classified into seven density classes: (1) <5, (2) 5.01–20, (3) 20.01–35, (4) 35.01–50, (5) 50.01–65, (6) 65.01–80, and (7) >80 stems ha^−1^. Nine species contributed 79.25% of the total density from the density class 7 which was due to the dominance of species *Carissa spinarum*, *Calpurnia aurea*, *Maytenus obscura*, *Grewia ferruginea*, *Capparis tomentosa*, *Premna schimperi*, *Rhus glutinosa*, *Pterolobium stellatum,* and *Croton macrostachyus*. From density class 1 *Stereospermum kunthianum*, *Ficus sycomorus*, *Ficus palmata*, *Dichrostachys cinerea*, *Acokanthera schimperi*, *Entada abyssinica*, *Ficussur*, *Piliostigma thonningii*, and *Bridelia micrantha* contributed only 0.23% of the total density.

According to the result, the densities of tree individuals of Wonjeta St Michael Church Forestwith DBH between 2.5 and 10 cm and between 10 cm and 20 cm, and with DBH > 20 cm were 1950.5 ha^−1^, 221.5 ha^−1^, and 30.5 ha^−1^, respectively. The ratio of density of individuals of Wonjeta St Michael Church Forest with DBH > 10 cm to DBH > 20 cm is very high (7.26). The comparison of the density ratio of the study site with other 4 forests' density ratio in Ethiopia is given by Table 2. The ratio a/b indicate that Wonjeta St Michael Church Forest has more tree in lower DBH classes than in the higher classes when compared to Gedo, Menna Angetu, Chilmo, and Menagesha Forest. The reasons behind these are geographical location, the nature of the forest, altitude variation, age of the forest, degree of conservation, and exposure to disturbance.

#### 3.3.2. DBH Distribution

**Figure 3:**
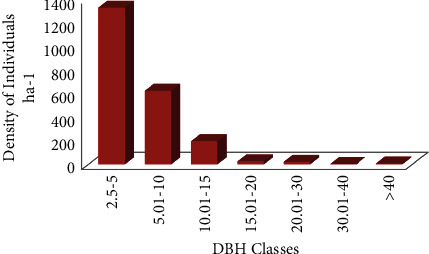
Distributions of woody plant individuals in the different DBH classes.

The distribution of plant species in different DBH classes is shown in Figure 3. Matured woody plants of the study area were classified into seven DBH classes. Class *A* = 2.5–5 cm, *B* = 5.01−10 cm, *C* = 10.01−15 cm, *D* = 15.01−20 cm, *E* = 20.01−30 cm, *F* = 30.01−40 cm, and *G* => 40 cm. Based on the result, the first class had the highest distribution of species density which was 1326.5 individuals ha^−1^ (60.23%), the second was 624 (28.33%) and the 3rd, 4th, 5th, and 6th takes 196.5 (8.92%), 25 (1.14%), 21 (0.95%), and 2.5 (0.11%), finally the last class takes 7 (0.32%), respectively.

The DBH class distribution of all individuals showed inverted J-shape distribution pattern which means that the majority of the species had highest number of individuals in the lowest diameter class. This means form the potential source of recruitment to successively increasing diameter classes that ensures sustained future regeneration of the forest if it will be properly managed. However, the density was decreased as DBH class increasing. This indicated that the predominance of small and medium sized individuals in the Forest such as *Carissa spinarum*, *Calpurnia aurea, Maytenusobscura*, *Grewiaferruginea,* and *Capparistomentosa*. This could be attributed to high rate of regeneration but poor recruitment in the forest, which might have been caused by unsustainable exploitation of woody species in the forest by the local people some years before maturity.

#### 3.3.3. Height Class Distribution

**Figure 4:**
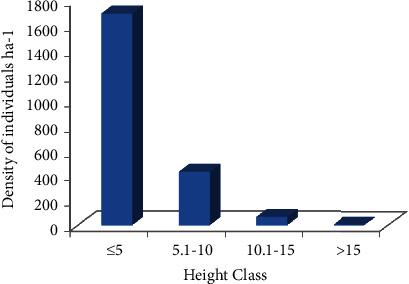
Distributions of woody plant individuals in the different height classes.

Individuals of woody species were classified into four height classes and their density ha^−1^ was shown on this basis (Figure 4). Based on the result, the height class distribution of woody plants of Wonjeta St Michael Church Forest showed a higher individual in the lowest height class, 1695.5 ha^−1^ (76.98%) are in ≤5 class height. As height increases from one class to the other the density of individuals were decreasing i.e., large proportion of woody plant species were distributed in the lowest height class. This clearly tells the dominance of small-sized individuals and the presence of high regeneration but lower recruitment and absence of matured individuals. It might be due to anthropogenic factors.

#### 3.3.4. Frequency

Based on the percentage frequency value, the result showed there are eight most frequent woody species in the study area ≥56% (Table 3).

#### 3.3.5. Population Structure

**Figure 5:**
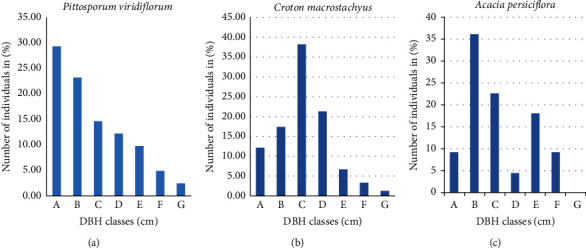
DBH distribution patterns of some group of woody species; *Pittosporumviridiflorum* (a), *Croton macrostachyus* (b), and *Senegalia persiciflora* (c) of representative woody species. DBH classes: *A* = 2.5–5 cm, *B* = 5.1–10 cm, *C* = 10.1–15 cm, *D* = 15.1–20 cm, *E* = 20.1–30 cm, *F* = 30.1–40 cm, and *G* => 40 cm.

In the present study, the general patterns of DBH distribution of the forest showed an inverted *J*-shape distribution pattern. However, some groups of individuals showed population dynamics and recruitment processes for a given species. The first type of pattern was inverted J-shape. It shows a high number of species in the lower DBH classes and reduction at the highest DBH classes. This pattern was exhibited by the species *Pittosporum viridiflorum* (Figure 5(a)). Such pattern shows normal or healthy structural pattern with good reproduction and recruitment capacity of a given species [40]. The second type of population pattern was bell shaped and is characterized by the species *Croton macrostachyus* (Figure 5(b)). It shows a fairly high number of individuals of the species in the middle DBH classes but lower numbers of individuals of the species in the lower and higher DBH classes. This species has poor recruitment potential which might be due to intense competition between the other species found in its surroundings and also were use of this tree for making fencing, charcoal and other purposes. The third type of population pattern is represented by *Senegalia persiciflora* (Figure 5(c)). In such pattern, the density of individuals increases with increasing DBH up to some point and then decreases with increasing DBH. The pattern continues with decreasing to some extent and increasing density as DBH increases. This population structure pattern showed irregular or zigzag type of distribution and is not healthy because of selective removal of the species for construction and fuel wood.

#### 3.3.6. Basal Area

The total basal area of woody species in St Michael Church Forest with DBH ≥ 2.5 cm and height ≥2 m was found to be 99.57 m^2^ha^−1^. Comparison of the basal area and density in the DBH classes of the study sites revealed that occurrence of more number of individuals in the first DBH classes 2.5–5 cm. However, their contribution to the basal area was very low. About 47.93% of the total basal area is distributed in the highest DBH Class (>20 cm) which was the presence of few but large sized individuals of the canopy trees which are *Ficus vasta*, *Sapium ellipticum*, and *Mimusops kummel*. The second highest basal area (28.5%) distribution is in DBH class 5.1–20 cm which was due to the constitution of species *Acacai alahai*, *Croton macrosachyus*, *Calpurnia aurea,* and *Rhus glutinosa*.

It was reported that BA provides a better measure of the relative importance of the species than simple stem count [41]. Thus, species with largest contribution to the basal area in St Michael Church Forest are *Vachellia lahai*, *Syzygium guineense*, *Calpurnia aurea*, *Premnaschimperi*, *Rhusglutinosa*, *Ficusvasta*, *Croton macrostachyus*, *Carissa spinarum*, *Grewiaferruginea,* and *Euclearacemosa.*

The basal area of St Michael Church Forest was compared with the basal areas of other five forests in Ethiopia (Table 4). Based on the result, the Basal area of St Michael Church Forest is much greater than Jibat, Menagesha, Gedo forest but smaller than Chilimo-Gaji forest and Belete forest. This may be due to variations in the conservation of the forests, exposure to deforestation, and geographical location of the forests.

#### 3.3.7. Dominant Plant Species

The total dominance of woody species in the forest was 49.78 m^2^ha^−1^. From 61 total plant species, the most dominant species in St Michael Church Forest was *Vachellia lahai* which contributed 16.13% followed by *Syzygium guineense* which contributed 10.35% and *Calpurnia aurea* which contributed 7.87%. Howevere, the least dominant species was *Ficussur*, *Combretum collinum*, *Piliostigma thonningii*, *Justica schimperiana*, *Entada abyssinica*, *Terminalia brownie*, *Stereospermum kunthianum*, *Helinumy stacinus*, *Ficus palmate*, *Acokanthera schimperi*, *Ficus sycomorus*, and *Dichrostachys cinerea* which contributed 0.1% of the total dominance. According to Feyera et al. [40], the high dominance and/or abundance of a few species in a forest could be attributed to a number of factors, such as the overharvesting of the desired species, disturbance factors, succession stage of the forest, and/or survival strategies of the species.

#### 3.3.8. Vertical Structure (Over Story) of St Michael Church Forest

The vertical stratification of the tree in the study area was examined using IUFRO classification scheme [23]. Based on this scheme, the top height was used for the vertical structure of tree. According to the result of the study trees with 2/3 of the top height (height above 24 m) represent upper story is 0.18% of the individuals; trees with height between 1/3 and 2/3 of the top height represent middle story contains 9.35% of the total individuals, and trees with height <1/3 of the top height represent lower story includes contains 90.47% of the individuals (Table 5). The highest tree distribution in the study area is the lower story class. This implies that the forest has been heavily influenced by the local anthropogenic activities through selective logging for fuel wood, construction, and illegal wood harvest for timber production. Currently, there were some long trees and short to medium individuals. The dominance of short-heighted individuals was the attribute of good regeneration but low recruitment.

#### 3.3.9. Regeneration Status

The result found the total densities of seedling, sapling, and mature plants of the forest were 2419.2 ha^−1^, 1737.6 ha^−1^, and 2202.5 ha^−1^, respectively. Out of the total analyzed tree and shrub fifteen (15) species were with no seedling and sapling, and they are put in the first priority class as they are represented by no individual and if no urgent action is taken they will be locally extinct those are *Acokantheraschimperi*, *Stereospermumkunthianum*, *Sapiumellipticum*, *Ficussycomorus*, *Syzyguimguineense*, *Ficusthonningi*, *Ficus palmate*, *Mimusops kummel*, *Ficusvasta*, *Brideliamicrantha*, *Dichrostachyscinerea*, *Erythrinaabyssinica*, *Albiziagummifera*, *Entadaabyssinica,* and *Ficus sur*.

The ratio analysis of woody species seedling to mature individuals in the forest gives that (1.10 : 1), seedling to sapling was (1.39 : 1), and sapling to mature (0.79 : 1). The result showed that there is more seedling than that of sapling and mature individuals implying the survival of seedling to reach sapling stage and according to Tiwari et al. [25], the forest is now in a fair regeneration. However, there are also species with no seedling and sapling that may be caused by due to the physical condition of their microhabitat and human impacts and need urgent measurement to be taken as they are in poor regeneration.

#### 3.3.10. Importance Value Index

The species in the forest were grouped in to five IVI classes based on their IVI values for conservation priority. Priority class 1 (IVI <1) should get 23 uppermost conservation priority since these species are at risk of local extinction. Those species with lower IVI values need high conservation efforts, while those with higher IVI values (IVI >14.1) need monitoring management. Based on their higher IVI value, there were nine (9) most dominant and ecologically most significant shrubs and trees species in St Michael Church Forest were *Carissa spinarum* (29.9), *Calpurina aurea* (25.63), *Vachellia lahai* (23.99), *Grewia ferruginea* (21.31), *Maytenus obscura* (20.78), *Premna schimperi* (19.0), *Rhus glutinosa* (17.02), *Capparis tomentosa* (15.47), and *Croton macrostachyus* (14.67). In contrast to this, *Phoenix reclinata*, *Phytolecadodecandra*, *Albizia gummifera*, *Acokanthera schimperi*, *Sapium ellipticum*, Senegalia venosa, *Erythrina abyssinica*, *Justica schimperiana*, *Sida schimperiana*, *Ficus palmate*, *Piliostigma thonningii*, *Millettia ferruginea*, *Hibiscus macranthus, and Stereospermum kunthianum* were species with low IVI value that need urgent feedback to regenerate. The possible reason for this could be either the selective cutting of these species by the local people or unfavorable microhabitat conditions. These indicate that as a whole conservation and management of the forest are required. Finally, the accepted manuscript has been deposited on a preprint server [44] as per the stated recommendation during the acceptance e-mail from Scienti<ca to the corresponding author.

## 4. Conclusion and Recommendations

### 4.1. Conclusion

From the result of species composition, coverage, and abundance of woody plants, the area will have a more complex and healthier community with a better environmental condition. This revealed that the forest can serve as in-situ conservation site. In this study, the result showed that the Forest is dominated by small-sized tree and shrub species, indicating that the Forest was seriously exploited and affected in the previous periods, but currently good regeneration is shown.

Based on structural description of diameter and height class distribution on St Michael Church Forest both DBH and height class shown similar trend, in that the density of tree and shrub species decrease with increasing DBH and height classes, which implied predominance of small-sized individuals in the lower classes than the higher diameter classes indicating good reproduction potential and rare occurrence of large individuals.

The analysis of frequency class in St Michael Church Forest showed that the higher percentage number of species in the lower class than in the higher frequency classes. This indicates that the Forest is floristically heterogeneous.

From the analysis of structures of some selected tree species in the study site, it can be concluded that some species such as *Vachellia sieberiana,* Senegalia venosa, *Acokanthera schimperi*, *Albizia gummifera*, *Entada abyssinica*, *Erythrina abyssinica*, *Ficus sycomorus*, and *Ficus vasta* have abnormal population structure with no or few individual at lower size classes and also there are species with no seedling and sapling as example *Sapiumellipticum*, *Ficusthonningii, Ficus palmate*, *Syzygiumguineense*, *Mimusops kummel*, *Stereospermumkunthianu*, *Dichrostachyscinerea*, *Erythrinaabyssinica*, *Entadaabyssinica,* and *Ficus sur*. These species need urgent conservation measures that will bring a healthy regeneration and consequently their sustainable use.

### 4.2. Recommendations

To conserve the Church Forest's genetic resources and to improve the natural diversity and structure of Church forest and to provide optimal support for development of Ethiopian upper Blue Nile basin for ecological, economical, and spiritual benefits, the following general recommendations were made:

- Complete ecological studies are vital such as, soil nutrient, soil seed bank, anthropogenic impact, and others of the forest, should be studied to identify the environmental factors responsible for the observed pattern.
- Analysis of IVI and species structure shows that some important tree species including *Sapium ellipticum*, *Ficus thonningii*, *Ficus palmate, Syzygium guineense*, *Erythrina abyssinica,* and *Entada abyssinica* are poor in regeneration and recruitment conditions, and this may be due to anthropogenic factors from the local community surrounding the area through cutting, continuous grazing, and traditional farming system; unless alternative measure and immediate attention are taken the species are in a heavy pressure.
- Governmental and nongovernmental institution should provide priority to the conservation of vegetation of the forest in general for its rich biodiversity area.
- Raising public awareness of the local community and introduction of participatory management programs should be encouraged and implemented. This will provide opportunity for local community to be involved in the management and conservation of the area and become beneficiaries.

## Figures and Tables

**Table 1:** The top 13 families with their corresponding number of genera and species.

No.	Family	Genera	%	Species	%
1	Fabaceae	10	18.87	14	21.54
2	Moraceae	1	1.89	6	9.23
3	Euphorbiaceae	4	7.55	4	6.15
4	Oleaceae	3	5.66	3	4.62
5	Lamiaceae	2	3.77	2	3.08
6	Combratiaceae	2	3.77	2	3.08
7	Myrtaceae	2	3.77	2	3.08
8	Apocynaceae	2	3.77	2	3.08
9	Acanthaceae	2	3.77	2	3.08
10	Celastraceae	1	1.89	2	3.08
11	Malvaceae	2	3.77	2	3.08
12	Solanaceae	1	1.89	2	3.08
13	Asteraceae	1	1.89	2	3.08
	Others	Each has one genera	1.89	Each has one species	1.54

**Table 2:** Comparison of tree densities with DBH between 10 and 20, and >20 cm of Wonjeta St Michael Church forest with other 4 forests in Ethiopia.

Forets	10 < DBH < 20 (a)	DBH > 20 (b)	a/b	Forest types	Source
Gedo	832	464	1.79	Dry afromontane	[31]
Menna Angeu	292.59	139.78	2.08	Dry afromontane	[37]
Chilmo	638	250	2.6	Dry afromontane	[38]
Menagesha	484	208	2.30	Dry afromontane	[39]
St. Micheal Church forest	221.5	30.5	7.26	Dry afromontane	Present study

**Table 3:** Frequency distribution of top 8 woody plant species of St. Michael Church Forest.

Number	Scientific name	Frequency in %
1	*Grewiaferruginea*	80
2	*Calpurnia aurea*	78
3	*Carissa spinarum*	76
4	*Maytenusobscura*	76
5	*Premnaschimperi*	74
6	*Croton macrostachyus*	70
7	*Rhusglutinosa*	62
8	*Vachellia lahai*	56

**Table 4:** Basal area (BA) comparison of St Michael Church Forest with other five afromontane forests (m^2^ha^−1^).

Forest	Basal area (m^2^ha^−1^)	Forest types	Source
Chilimo-Gaji	454.52	Dry afromontane	[42]
Belete	103.5	Moist evergreen montane	[43]
Jibat	49.8	Humid afromontane	[41]
Gedo	35.45	Dry afromontane	[31]
Menagesha	36.1	Dry afromontane	Tamrat [39]
Present study site	99.57	Dry afromontane	Present study (2020)

**Table 5:** Over story classification of the forest.

Vertical stratification of the trees	Number of individuals	Individuals in (%)
Upper story	8	0.18
Middle story	412	9.35
Lower story	3985	90.47

## Data Availability

The data sets used and/or analyzed in the study are available within the article.
